# Genome-Wide Survey of Pseudogenes in 80 Fully Re-sequenced *Arabidopsis thaliana* Accessions

**DOI:** 10.1371/journal.pone.0051769

**Published:** 2012-12-13

**Authors:** Long Wang, Weina Si, Yongfang Yao, Dacheng Tian, Hitoshi Araki, Sihai Yang

**Affiliations:** 1 State Key Laboratory of Pharmaceutical Biotechnology, School of Life Sciences, Nanjing University, Nanjing, China; 2 Eawag, Swiss Federal Institute of Aquatic Science and Technology, Center of Ecology, Evolution and Biogeochemistry, Kastanienbaum, Switzerland; Universität des Saarlandes, Germany

## Abstract

Pseudogenes (*Ψs)*, including processed and non-processed *Ψs*, are ubiquitous genetic elements derived from originally functional genes in all studied genomes within the three kingdoms of life. However, systematic surveys of non-processed *Ψs* utilizing genomic information from multiple samples within a species are still rare. Here a systematic comparative analysis was conducted of *Ψs* within 80 fully re-sequenced *Arabidopsis thaliana* accessions, and 7546 genes, representing ∼28% of the genomic annotated open reading frames (ORFs), were found with disruptive mutations in at least one accession. The distribution of these *Ψs* on chromosomes showed a significantly negative correlation between *Ψs*/ORFs and their local gene densities, suggesting a higher proportion of *Ψs* in gene desert regions, e.g. near centromeres. On the other hand, compared with the non-*Ψ* loci, even the intact coding sequences (CDSs) in the *Ψ* loci were found to have shorter CDS length, fewer exon number and lower GC content. In addition, a significant functional bias against the null hypothesis was detected in the *Ψ*s mainly involved in responses to environmental stimuli and biotic stress as reported, suggesting that they are likely important for adaptive evolution to rapidly changing environments by pseudogenization to accumulate successive mutations.

## Introduction

Pseudogenes (*Ψ*s) are found in all studied genomes within the three kingdoms of life. They are ubiquitous genetic elements derived from originally functional genes after mutational inactivation, such as premature stops or frameshift mutations [1]. Therefore, *Ψ*s are defined as “defunct” genes or “junk” DNA as they have lost their ability to code functional products [1]–[5]. A *Ψ* can be generated from a single functional gene or a duplicated gene copy. *Ψ*s are called processed if they originated from retro-transposition and non-processed if they are from DNA duplication events [6].

Processed *Ψ*s are common in mammalian species but much less abundant in plant species [7]. For example, approximately 5000 and 8000 processed *Ψ*s have been detected in mouse and human genomes, respectively [8], [9], whereas only 411 processed sequences and 376 processed *Ψ*s were identified in the *Arabidopsis thaliana* genome (c.a. 1.61 and 1.47% of the *Arabidopsis* genes, respectively) [7]. The processed genes are randomly distributed in the *Arabidopsis* genome and tend to have originated from genes with high copy numbers but not from highly expressed genes. In addition, evolutionary and expression analyses suggest that a large number of *Ψ*s in *Arabidopsis* and rice genomes had been subjected to purifying selection for substantial periods of time before pseudogenization, and that gene families involved in environmental stress responses have a significant excess of *Ψ*s [10].

Since first discovered in 1977 [1], [11], *Ψ*s have been studied in various genomes among different kingdoms [2]. Nevertheless, systematic surveys of non-processed *Ψ*s utilizing genomic information from multiple samples in a species are still rare. The rapid development of nucleotide sequencing technology has provided a unique opportunity to explore the origin and fate of *Ψ*s between related species or between populations within a species [12]. For example, a comparative analysis of *Ψ*s within the fully sequenced genomes of eight yeast species has shown that most of the *Ψ*s originated from mutational degradation of gene copies after species-specific duplications, and that *Ψ* formation contributed to the rapid genome evolution through gene duplications and losses in yeasts [2]. Furthermore, by resequencing 20 diverse strains in *Arabidopsis*, Clark et al. [13] showed that 1614 genes have large-effect single nucleotide polymorphisms (SNPs) that are expected to affect the integrity of encoded proteins, of which 1227 introduced premature stop codons, 156 altered initiation methionine residues, and 435 led to nonfunctional splice donor or acceptor sites. Interestingly, another re-sequencing project in 18 *A. thaliana* natural accessions predicted approximately one-third of protein-coding genes to be disrupted in at least one accession, but most genes were restored through re-annotation of each genome [14]. A genome-wide study of SNP variations in 20 diverse rice varieties also showed that approximately 2.7% of the rice genes contain large-effect SNPs that presumably affect the integrity of encoded proteins [15]. Another genome-wide survey in 517 rice landraces detected large-effect SNPs in 3039 out of 25409 annotated genes with transcript support (∼12.0%) [16].

As *Ψ*s are ubiquitous genetic elements derived from functional genes after mutational inactivation, their characterization is important for understanding genome dynamics and evolution. In this study, we addressed the following questions: (i) What are the dynamics of pseudogenization from functional genes in or between their populations within a species? (ii) What is the distribution of *Ψ*s over the whole genome, and are there regional effects on these *Ψ*s? (iii) Is there a functional preference for the pseudonization of genes on the whole genome scale? (iv) Does natural selection play an important role in generating these *Ψ*s? To address these questions, we utilized the high-quality, fully re-sequenced data from 80 *A. thaliana* accessions reported by Cao et al. [17]. The advent of these genome-wide data sets with individuals from many populations across a wide geographic range has allowed us to systematically investigate the genome-wide patterns of the *Ψ*s in the model organism and their chromosomal organization among the world-wide accessions.

## Materials and Methods

### Identification of Ψs

The *Arabidopsis* Col-0 ecotype genome assembly (TAIR9) was downloaded from the TAIR (data downloaded from ftp://ftp.arabidopsis.org/Genes/TAIR9_genome_release/) website and used as a reference genome to detect mutations [18]. Comparisons were then made between the reference genome and the 80 re-sequenced *A. thaliana* accessions (data from the 1001 Genome Project, http://1001genomes.org/data/MPI/MPICao2010/) [17] using custom PERL scripts (scripts are available for download on the website http://gattaca.nju.edu.cn/scripts/Pseudogenes/). Only the high quality annotations of genome differences by the 1001 Genomes Projects (filtered_reference, filtered_variantion and insertion) were used to ensure the reliability of the results, while the inaccessible regions caused by zero coverage or no possible call were assumed to remain the same as the reference genome so that artificial errors would be minimized in the analysis.

*Ψ*s were defined based on the presence of a frameshift mutation or a premature stop codon in the open reading frame (ORF). A frameshift mutation was detected if indels (insertions or deletions) caused the number of nucleotides to not be evenly divisible by three in the coding region. In the case that the indels resulted in an evenly divisible number of nucleotides, three adjacent indels were treated as a set from start to end. Once an interval larger than 300 bp between the first and last indel was found in any set, this mutation was also added to the frameshift mutation, as it would largely affect the translation of the coding sequence (CDS). A nonsense mutation was detected if a premature stop codon was found in the ORFs. Since we did not examine the functionality of each gene and allele, we used multiple criteria based on the location of the disruptive mutations to define *Ψ*s (1/3, 2/3 or 3/3 of the ORF, see Results). In addition, if there are multiple transcripts in the gene locus, only the first transcript was used.

The protein-coding genes already annotated as *Ψ*s or transponsons were excluded from the database in this study. The remaining annotated ORFs in the genome sequence in Col-0 (Col-ORFs) were used as a reference to categorize genes into *Ψ* loci and non-*Ψ* loci, based on the presence and absence of any disruptive mutation, respectively, in the 80 re-sequenced genomes. Alleles were either considered disrupted as defined above or intact in the *Ψ* loci of the 80 re-sequenced genomes. If multiple disruptive mutations were found in one ORF, the upper-most disruptive mutation was used for practical categorization of the *Ψ* loci as shown in Table 1. Given that a *Ψ* can be first created by a downstream disruptive mutation, this is a technical categorization rather than an evolutionary one (i.e. the evolutionary origin of pseudogenization may be different). To understand its evolutionary process, therefore, a subsample of *Ψ* loci, each carrying only one disruptive mutation in its ORF, was also extracted and examined for potential changes in the *Ψ* distribution. The gene density estimated as the gene numbers in a 1-Mb region.

**Table 1:** Number of *Ψ* loci in 80 re-sequenced *A. thaliana* accessions.

Disabling mutations	Relative position of the disabling mutations comparing with their intact alleles in Col-0			
	0–1/3 a	1/3–2/3 b	2/3–1 c	Total
Frameshift	2676	2302	2611	5750
Premature stop	1866	1769	2052	4238
Total	3836	3457	4036	7546
*Ψ*s number (0–2/3)	5699			

a disabling mutations occurring in the first one-third of the ORFs.

b disabling mutations occurring in the second one-third of the ORFs.

c disabling mutations occurring in the last one-third of the ORFs.

Note: These are the numbers of *Ψ*s identified in at least one accession in the 80 re-sequenced *Arabidopsis* accessions.

### Classification of Clusters and Families

To investigate the influence of gene density, a *Ψ* cluster was classified when two *Ψ*s were separated by less than ten genes in each chromosome. The *Ψ* loci that were not grouped into any *Ψ* cluster were considered *Ψ* singletons.

All-versus-all local BLASTN [19] with an e-value 10e–40 was applied to detect homologue families among the TAIR9 annotated CDS. The gene with no hits other than itself was considered a single gene. Other multi-hits genes were classified into families with an identity ≥70% and coverage ≥50%. After classification of the families, a pairwise local alignment with ClustalW2 [20] was implemented in each family. A new identity value was then obtained by dividing the nucleotide identity by the total number of nucleotides compared in each aligned pair, and the maximum value was taken as the identity for each family in subsequent analyses.

To investigate regional differences in the frequency of *Ψ*s, *Ψ* loci were compared between the centromere and telomere regions. A total of 4 Mb encompassing a centromere for a centromere region and 4 Mb from each tip of the chromosome for a telomere region were used in this analysis.

### Evolutionary Rate and Functional Analysis

Synonymous and nonsynonymous substitution rates (*Ks* and *Ka*, respectively) were calculated based on the equations of Nei and Gojobori (1986) with the Jukes and Cantor model (1969) within and between the disrupted alleles and its corresponding intact alleles for each *Ψ* locus. As one locus can have different disruptive mutations in different accessions, only the disrupted allele with the highest frequency was used, and the pseudo-alleles with low frequency were excluded in the subsequent analyses.

Functional domains were identified by searching the TAIR9 protein sequences against the Pfam library of HMMs, the search was implemented locally using the ‘pfam_scan.pl’ script [21] against the Pfam database release 24 (downloaded from ftp://ftp.sanger.ac.uk/pub/databases/Pfam) with default options. Information on the regional distribution was obtained from the 1001Genomes website above. The phylogenetic tree was generated according to the symbolic sequences constructed by using ‘1’ in place of a non-*Ψ* locus in each ecotype and ‘0’ in place of a *Ψ* locus in each ecotype by using the PHYLIP package v3.6 based on the neighbor-joining method.

## Results

### Identification of Ψs in the 80 Sequenced A. thaliana Accessions

Compared with the 27133 annotated ORFs in *A. thaliana* reference accession Col-0, disruptive mutations were detected in 7546 genes (28.0%, data available at http://gattaca.nju.edu.cn/data/Pseudogenes/) defined as *Ψ*s in at least one accession among the 80 re-sequenced accessions (Table 1). If the more stringent criteria was used that the first disruptive mutation should be located in the first 1/3 or 2/3 of the annotated ORFs, then the numbers of *Ψ*s should be 3836 (14.1%) or 5699 (21.0%), respectively, suggesting a large number of *Ψ*s maintained in the *Arabidopsis* populations. In addition, the disruptive mutations are more likely to occur at the end of the genes (Table 1; Chi-square test, *P*<0.01). Most recently, 1939 annotated protein-coding genes with little evidence of expression were identified as possible *Ψ*s by Yang and his colleagues [22]. Interestingly, 1230 of these genes (1230/1939 = 63.4%) with disruptive mutations were detected at least in one accession in our study.

**Figure 1:**
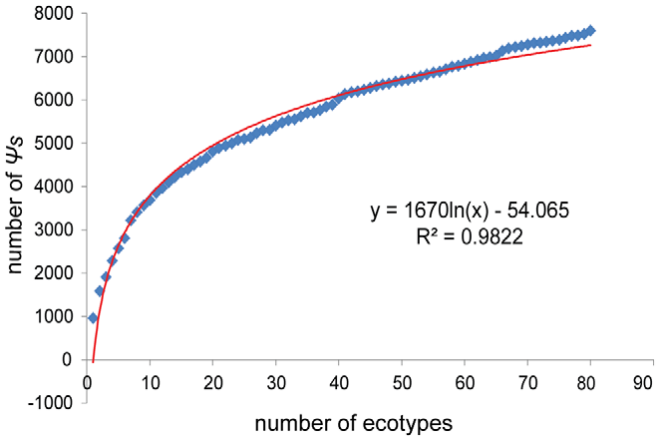
Increase in number of *Ψ*s relative to 80 Arabidopsis accessions sampled.

The average number of *Ψ*s in each accession was 930±68 (∼3.4% of the annotated ORFs in Col-0), ranging from 794 in accession Rue3-1-31 to 1209 in accession Don-0 (Table S1). To further evaluate the distribution of *Ψ*s in the wild populations, the relationship between the number of *Ψ* loci and the sample size (i.e. number of accessions) was analyzed. Essentially, the number of *Ψ* loci increased logarithmically as the number of sampled accessions increased (Figure 1). Using a regression approach, a formula (Y = 1670ln(X)-54.06, *r^2^* = 0.982) was obtained to predict the number of *Ψ* loci (Y) from the sample size (X). According to this formula, 11786 *Ψ* loci would be identified if 1200 genetically distinct accessions exist in the wild *A. thaliana* populations, as indicated by Weigel and Mott [23]. If true, our results indicate that at least one disrupted allele was present for nearly half of the genes (∼43.4%) in the wild populations of *A. thaliana*.

Among the 80 re-sequenced accessions, the average frequency of disrupted alleles was 5.99 at each *Ψ* locus (7.5%). The frequency distribution of these *Ψ*s has shown that frameshift alleles are slightly larger than these of premature alleles (Figure S1). A total of 4240 *Ψ* loci (55.8%) were shared among at least two accessions and approximately 86% of the *Ψ* loci were shared from 1 to 10 accessions (Table S2).

### Regional Distributions of Ψ Loci in the Genome

**Figure 2:**
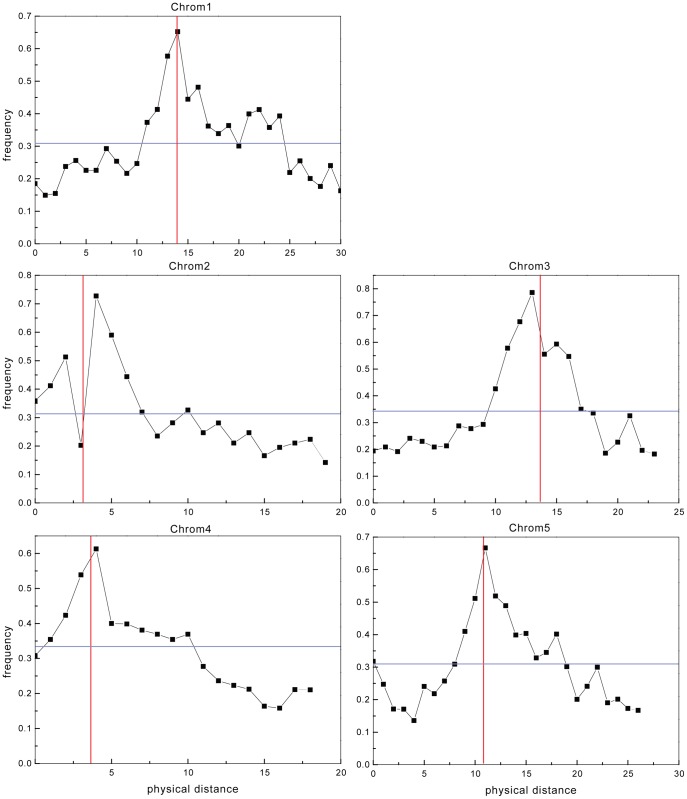
*Ψ* distribution along Arabidopsis chromosomes. The X-axis represents the physical distance (Mb) along the indicated chromosomes. The Y-axis represents the frequency of *Ψ*s in the region.

The distribution of *Ψ* loci on the five chromosomes showed that the proportion of *Ψ* loci among the ORFs (*Ψs*/ORFs) was similar among the chromosomes (Table S3). However, when the proportions of *Ψ* loci were calculated in 1-Mb sliding window regions along the genome, the distribution of *Ψ*/ORF along all chromosomes was found to be strongly influenced by their centromeric location (Table S4 and Figure 2). The major *Ψs*/ORFs peaks occurred around the centromere regions, whereas the lower *Ψs*/ORFs peaks were found in the telomere regions. The centromere regions showed roughly three- or four-fold higher *Ψs*/ORFs than the telomere regions (Table S4), which is consistent with the most recent report that the pericentromeric region is rich in pseudogenes [24].

**Figure 3:**
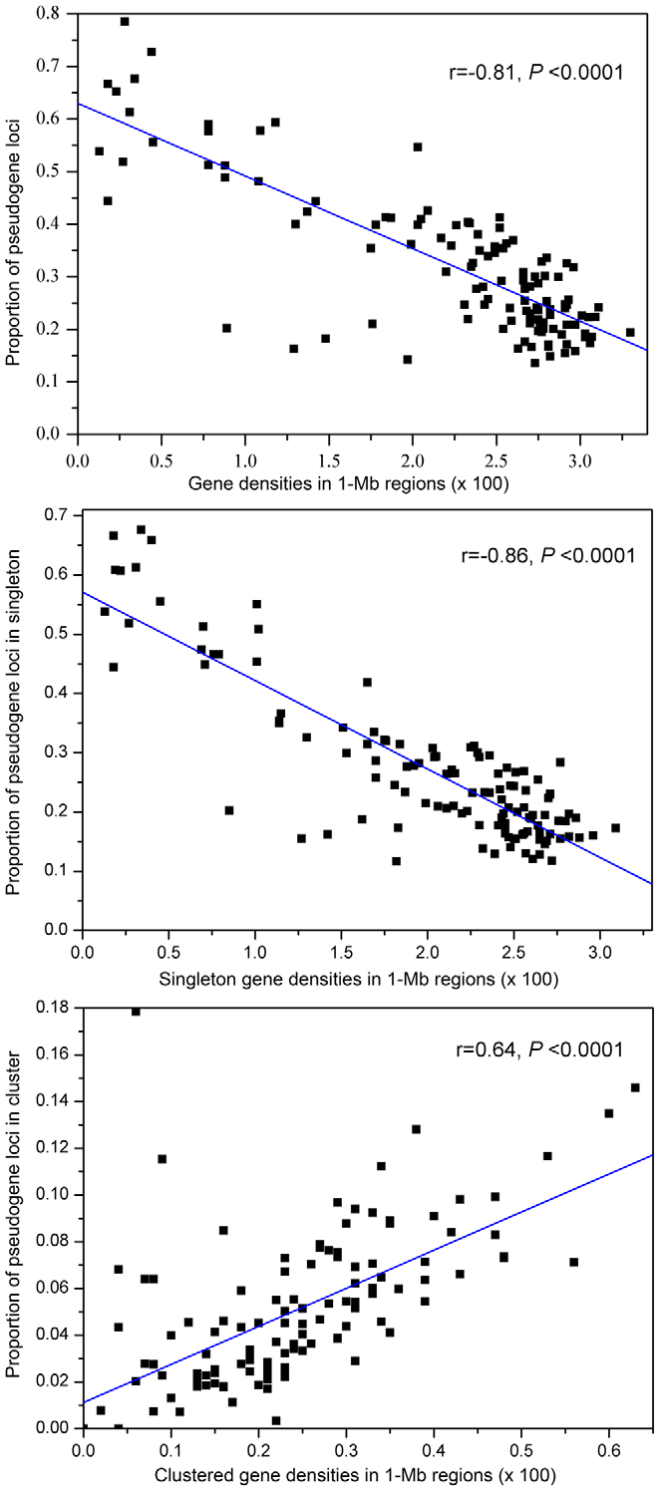
Relationships of *Ψ* distribution and gene density in *A. thaliana* chromosome regions. The gene density for each dot is counted by all genes in a 1-Mb region in reference genome of Col-0 and only protein-encoding genes were counted, including predicted and hypothetical genes, but excluding genes related to transposons. (A) A significantly negative correlation was detected between the proportion of pseudogene loci and gene densities, (B) The frequency of pseudogene loci located in singleton loci was also significantly negatively correlated with the singleton gene densities. (C) A significantly positive correlation was found between the frequency of clustered pseudogene loci and their gene densities.

Previous results have shown that the *A. thaliana* centromere regions have lower local gene densities than chromosome telomere regions [18], then we asked whether the gene desert regions may have a higher proportion of *Ψ* loci. To test this hypothesis, the relationship between the proportions of *Ψ* loci with local gene densities in the same regions was examined. As expected, a significantly negative correlation was observed between *Ψs*/ORFs and local gene densities (r = −0.81, *P*<0.0001; Figure 3A).

On the other hand, gene duplication plays an important role in providing raw materials for the evolution of genetic diversity. Due to the functional redundancy, many duplicated genes accumulate disruptive mutations and become *Ψ*s. As expected, a significantly positive correlation was found between the duplicated *Ψs* and the density of duplicated genes (r = 0.64, *P*<0.0001; Figure 3C). However, no significant correlation was detected between the number of duplicated *Ψs* and gene densities (*P* = 0.47), while the proportions of singleton *Ψs* shared a significantly negative correlation with the singleton gene densities (r = −0.86, *P*<0.0001; Figure 3B), suggesting that the *Ψ* singletons may play a major role in the relationship between the proportions of *Ψs* and gene densities.

To further evaluate the characteristics of *Ψ*s, allele frequency, the length of CDS in reference genome, exon numbers and GC content were compared between centromere and telomere regions (Table 2 and Table S4). On average, using the intact CDSs in reference genome, *Ψ* loci in centromere regions showed a significantly shorter CDS, fewer exon number, lower GC content and higher frequency of pseudogenization among the 80 re-sequenced accessions than those of *Ψ* loci in telomere regions (*P*<0.001; Table 2). On the other hand, compared with the non-*Ψ* loci located in the same regions, the *Ψ* loci also had a significantly shorter CDS length, fewer exon number and lower GC content (*P*<0.001; Table 2), except in the case of CDS length within telomere regions (Table 2). In addition, the average length of these *Ψ*s identified in our study is significantly longer than that of processed *Ψ*s (*P*<0.01) [7]. These results indicate that the gene*s*, located in regions with lower gene densities, with shorter CDS length, fewer exon number and lower GC content, are more likely to become *Ψ*s in the *A*. *thaliana* populations. The *Ψ*s with less GC content could indicate the result of biased gene conversion which has been confirmed recently [28].

**Table 2:** Characteristics of *Ψ*s in telomere and centromere regions.

Chromosomes		Average frequency of *Ψ*s in the 80 resequenced accessions			Length (bp)			Exon No.			GC%		
		Centro	Telo	*t*-test, *P ^d^*	Centro	Telo	*t*-test, *P ^d^*	Centro	Telo	*t*-test, *P ^d^*	Centro	Telo	*t*-test, *P ^d^*
1	*Ψ*s ^a^	0.124	0.082	9.8E-06, ***	943	1363	9.8E-06, ***	4.2	4.96	0.05 *	0.42	0.44	2.6E-05, ***
	*Reference* ^b^	–	–	–	1141	1287	0.065	5.51	5.74	0.32	0.44	0.45	0.0001, ***
	t-test, *P^ c^*	–	–	–	7.7E-06	0.14	–	6.7E-05	0.005	–	2.4E-05	1.8E-10	–
2	*Ψ*s	0.182	0.091	9.8E-06, ***	834	1178	0.001, ***	3.44	4.32	0.05 *	0.43	0.44	0.02, *
	Reference	–	–	–	932	1193	8E-04, ***	4.86	5.24	0.27	0.446	0.154	0.02, *
	t-test, *P*	–	–	–	0.16	0.43	–	0.02	0.01	–	0.001	2E-06	–
3	*Ψ*s	0.179	0.080	9.8E-06, ***	877	1257	2.1E-06, ***	3.5	4.22	0.028 *	0.43	0.44	0.26
	Reference	–	–	–	998	1214	0.03, *	4.2	5.54	0.009, **	0.44	0.45	0.001, ***
	t-test, *P*	–	–	–	0.14	0.18	–	0.11	4E-06	–	0.47	1E-18	–
4	*Ψ*s	0.144	0.075	9.8E-06, ***	961	1281	0.002, **	3.91	4.52	0.12	0.43	0.44	0.01, **
	Reference	–	–	–	1144	1272	0.03, *	5.6	5.6	0.46	0.43	0.45	3E-08, ***
	t-test, *P*	–	–	–	0.03	0.46	–	4E-04	0.008	–	0.02	5E-05	–
5	*Ψ*s	0.138	0.071	9.8E-06, ***	801	1270	2.4E-08, ***	3.43	5.18	7.1E-05, ***	0.44	0.44	0.34
	Reference	–	–	–	1099	1227	0.05, *	5.3	5.4	0.44	0.44	0.45	0.001, ***
	t-test, *P*	–	–	–	0.001	0.21	–	0.001	0.05	–	0.22	1E-11	–
Average	*Ψ*s	0.153	0.079	9.8E-06, ***	885	1216	3.6E-22, ***	3.70	4.54	5.2E-07, ***	0.43	0.44	0.001, ***
	Reference	–	–	–	1064	1240	9E-07	5.18	5.53	0.06	0.44	0.45	7E-14
	t-test, *P*	–	–	–	5E-05	0.10	–	3E-08	5E-08	–	1E-05	4E-47	–

Centro, centromere; Telo, telomere; ^a^ allelic *Ψ* loci, intact ORFs in Col-0 were used; ^b^ all allelic non-*Ψ* loci in these regions; ^c^ comparison between*Ψ*s and reference genes; ^d^ comparison between telomere and centromere regions; *P<0.05, **P<0.01, ***P<0.001.

### Distinct Regional Patterns of Nucleotide Substitutions in Ψ loci

Previous studies have shown that there is a higher level of nucleotide diversity in centromere regions than in chromosome arm regions in *Arabidopsis* [13], [15], [25]–[27]. A similar pattern of nucleotide diversity was also found in *Ψ* loci among the 80 accessions. Significantly higher regional nucleotide diversities were observed in the centromere than in the telomere both in the disrupted alleles and in the intact alleles in *Ψ* loci (Table 3). In synonymous sites, the rate of substitutions in the centromere region was on average 1.5∼3.5 times higher than in the telomere region, and it was 2.0∼4.3 times greater in nonsynonymous sites. A similar pattern was also found between telomere and centromere regions in non-*Ψ* loci (*t*-test, *P*<0.001). However, in the centromere regions, the average *Ka* in non-*Ψ* loci (0.0026) was approximately 3.2-fold lower than that in *Ψ* loci (0.0082, *P* = 5E−68), whereas the average *Ks* in non-*Ψ* loci (0.011) was slightly but significantly lower than that in *Ψ* loci (0.013, *P*<0.001; Table 3). These results suggest that the distinct pattern of nucleotide diversity between centromere and telomere regions can be found among any type of genes, but it is most pronounced in nonsynonymous substitutions in *Ψ* loci. More recently, Yang and colleagues have shown that unexpectedly high gene conversions near centromeres may explain why Arabidopsis has unusually high diversity near centromeres [28], which might also be a reasonable cause for *Ψs* in these regions.

**Table 3:** Comparison of nucleotide substitution rates between telomere and centromere regions.

Chromosomes		Average *Ka*						Average *Ks*						Average *Ka/Ks*					
		Disrupted ^e^			Intact ^f^			Disrupted			Intact			Disrupted			Intact		
		C(%)	T(%)	*P ^d^*	C (%)	T (%)	*P ^d^*	C(%)	T(%)	*P ^d^*	C(%)	T(%)	*P ^d^*	C	T	*P ^d^*	C	T	*P ^d^*
1	*Ψ*s ^a^	0.83	0.23	2E-10	0.75	0.28	7E-15	1.23	0.50	2E-07	1.35	0.69	2E-06	0.67	0.50	0.004	0.55	0.42	0.022
	non*-Ψ*s ^b^	–	–	–	0.28	0.13	3E-06	–	–	–	1.42	0.58	3E-10	–	–	–	0.20	0.22	0.22
	t-test, *P^ c^*	–	–	–	1E-13	0.002	–	–	–	–	0.31	0.07	–	–	–	–	0.001	0.03	–
2	*Ψ*s	0.99	0.23	3E-15	0.94	0.26	4E-24	1.38	0.48	3E-08	1.44	0.62	4E-12	0.71	0.49	0.012	0.65	0.42	0.027
	non*-Ψ*s	–	–	–	0.19	0.12	0.03	–	–	–	0.73	0.52	0.009	–	–	–	0.27	0.24	0.23
	t-test, *P*	–	–	–	7E-25	7E-10	–	–	–	–	3E-08	0.64	–	–	–	–	0.043	0.05	–
3	*Ψ*s	1.02	0.27	1E-13	0.94	0.29	7E-20	1.68	0.47	7E-11	1.48	0.60	2E-11	0.61	0.60	0.56	0.64	0.48	0.04
	non*-Ψ*s	–	–	–	0.38	0.12	2E-06	–	–	–	1.13	0.56	2E-06	–	–	–	0.33	0.22	0.34
	t-test, *P*	–	–	–	8E-12	7E-19	–	–	–	–	0.02	0.11	–	–	–	–	0.03	0.01	–
4	*Ψ*s	0.65	0.22	8E-06	0.70	0.27	1E-12	1.09	0.53	2E-04	1.25	0.73	2E-05	0.60	0.42	0.023	0.56	0.38	0.07
	non*-Ψ*s	–	–	–	0.29	0.12	2E-08	–	–	–	1.30	0.50	2E-14	–	–	–	0.22	0.23	0.33
	t-test, *P*	–	–	–	1E-11	3E-07	–	–	–	–	0.37	0.006	–	–	–	–	0.004	0.05	–
5	*Ψ*s	0.72	0.31	3E-08	0.69	0.33	6E-10	1.20	0.54	3E-05	1.14	0.75	3E-04	0.60	0.42	0.07	0.60	0.46	0.008
	non*-Ψ*s	–	–	–	0.22	0.15	0.004	–	–	–	1.06	0.63	5E-05	–	–	–	0.21	0.23	0.69
	t-test, *P*	–	–	–	1E-13	6E-20	–	–	–	–	0.28	0.005	–	–	–	–	0.002	0.013	–
Average	*Ψ*s	0.85	0.25	7E-44	0.81	0.29	1E-71	1.33	0.49	7E-30	1.34	0.68	4E-35	0.64	0.51	5E-04	0.60	0.43	0.001
	non*-Ψ*s	–	–	–	0.26	0.13	2E-16	–	–	–	1.11	0.57	6E-28	–	–	–	0.24	0.23	0.86
	t-test, *P*	–	–	–	5E-68	9E-63	–	–	–	–	3E-04	4E-06	–	–	–	–	0.003	0.009	–

C, centromere regions; T, telomere regions; ^a^ *Ψ* loci; ^b^ non-*Ψ* loci; ^c^ *t-*test between*Ψ* and non*-Ψ* loci; ^d^ *t*-test between telomere and centromere regions; ^e^ disrupted alleles, in which nucleotide substitution rates were calculated; ^f^ intact alleles; *P<0.05, **P<0.01, ***P<0.001.

**Figure 4:**
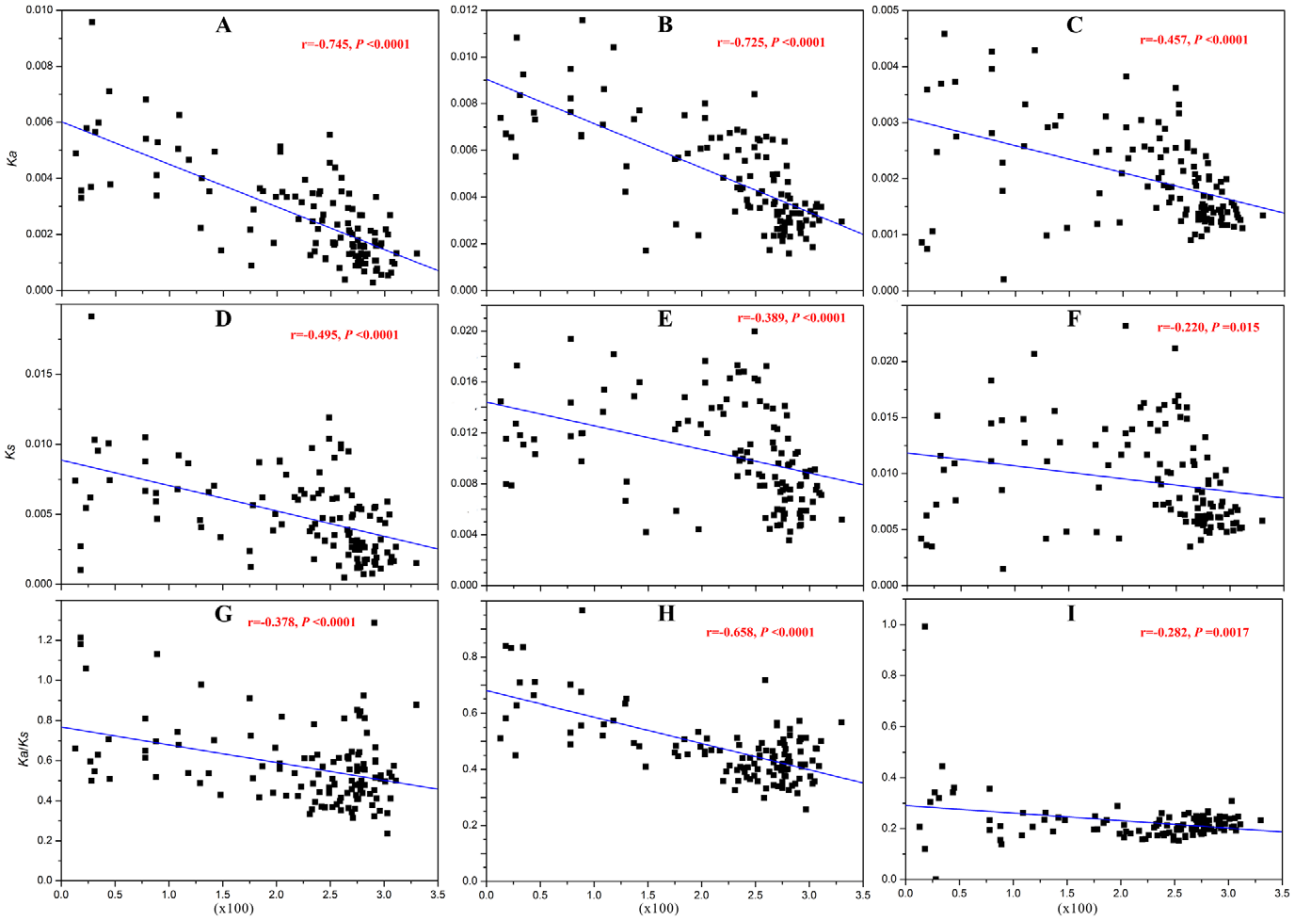
Relationships of non-synonymous (*Ka*) and synonymous (*Ks*) substitutions or their ratios (*Ka/Ks*) with gene densities. In all plots, the *X-axis* represents the gene density estimated as the gene numbers in a 1-Mb region. The counts were for protein-encoding genes only, including predicted and hypothetical genes, but excluding genes related to transposons. The *Y-axis* represents the nucleotide substitutions. (A) Non-synonymous substitutions among disrupted alleles within *Ψ* loci; (B) Non-synonymous substitutions among intact alleles within *Ψ* loci in the 80 *A. thaliana* accessions. (C) Non-synonymous substitutions among intact alleles within non-*Ψ* loci. (D) Synonymous substitutions between disrupted alleles within *Ψ* loci. (E) Synonymous substitutions among intact alleles within *Ψ* loci. (F) Synonymous substitutions among intact alleles within non-*Ψ* loci. (G) Average *Ka/Ks* among disrupted alleles within *Ψ* loci. (H) Average *Ka/Ks* among intact alleles within *Ψ* loci. (I) Average *Ka/Ks* among intact alleles within non- *Ψ* loci.

Since a significantly negative correlation was observed between the proportions of *Ψ* loci and the local gene densities (*P*<0.001; Figure 3A), it was of interest to know whether the rate of nucleotide substitutions of *Ψ* loci also negatively correlates with local gene densities. Indeed, a strong negative correlation between *Ka* and the gene densities was detected in *Ψ* loci (*r* = −0.75, *P*<0.001; Figure 4A and B), and a slightly less negative correlation was found in non-*Ψ* loci (*r* = −0.46, *P*<0.001; Figure 4C). *Ks* was relatively weakly correlated with the local gene densities in both *Ψ* and non-*Ψ* loci (Figure 4D–F). These results further indicate that the distribution of polymorphisms across the genome is markedly nonrandom, and that the differences in local gene density may influence the regional proportion of *Ψ* loci and substitution rates in them.

The distribution pattern of *Ψ* loci could have been attributed to artificial errors in the re-sequencing technology. To evaluate this possibility, 30 *Ψ* loci in the average of 10 accessions per locus were randomly selected from the database, and their sequences were confirmed by Sanger sequencing (see Methods). A total of 237 kb from 297 sequences were obtained, in which 1117 mutations (124 disabling mutations) were expected compared with the Col-0 genome. The sequence data recovered 1103 (98.7%) mutations and 121 (97.6%) disabling mutations, suggesting that the artificial errors were rare and could not account for the *Ψ* distribution observed in this study.

### Relaxation of Selective Pressures in Ψ Loci

It has been reported that the high nucleotide diversity of genes in the centromere region is not due to lack of selective constraint but due to too few targets for purifying or positive selection in the centromere regions [27]. According to this hypothesis, the average *Ka*, *Ks* or *Ka/Ks* in *Ψ* loci was expected to be roughly equal to that in non-*Ψ* loci when comparing their intact alleles between *Ψ* and non-*Ψ* loci in the same regions. However, if the high nucleotide diversity of the centromere genes was due to the relaxation of selective pressures in the *Ψ* loci, it was expected that (i) the average *Ka* or *Ka/Ks* in *Ψ* loci should be larger than that in non-*Ψ* loci (ii) and *Ks* in *Ψ* loci would be roughly equal to that in non-*Ψ* loci. Interestingly, either in centromere or telomere regions, (i) the average *Ka* or *Ka/Ks* in *Ψs* loci was significantly larger than that in non-*Ψ* loci (*P*<0.005); (ii) *Ks* in *Ψs* loci was slightly larger than that in non-*Ψ* loci (Table 3), suggesting that these *Ψ* loci might be undergoing relaxed selection.

Among the 4260 *Ψ*s loci shared by at least two accessions, the nucleotide divergences (*D_xy_*) between disrupted and intact alleles were significantly larger than the nucleotide diversity in each allelic group (Table 4; paired *t*-test, *P*<0.001). In addition, the diversity (π) between *Ψ* alleles was significantly positively correlated with the increasing frequency of *Ψ*s in the 80 accessions (Table 4; *P*<0.05). Because most *Ψ* loci had a low frequency (shared among 2–10 accessions), nucleotide substitutions were counted only in these low-frequency *Ψ* loci. Figure S2A shows that both *Ka* and *Ks* were significantly positively correlated with the frequencies of the disrupted alleles, but this trend was weaker in their corresponding intact alleles (Figure S2B). Moreover, these disrupted alleles had a significantly larger *Ka/Ks* than that in their intact alleles (paired *t*-test, *P*<0.001). All these results also suggest a signature of relaxed selective constraint after their pseudogenization.

**Figure 5:**
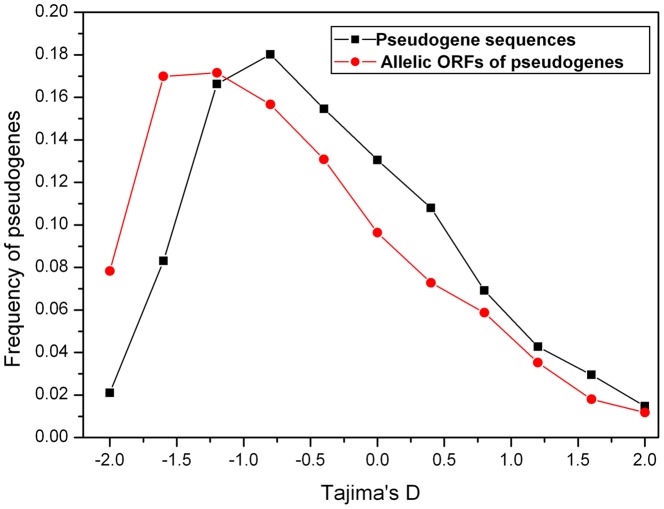
Distribution of Tajima’s *D* statistic across *Ψ* loci.

**Table 4:** Relationship between the frequency of disrupted alleles in the 80 *A. thaliana* accessions and their genetic diversities.

Frequency of disrupted alleles	Among disrupted alleles				Among intact alleles				Between disrupted and intact alleles			
	π (%)	*Ka* (%)	*Ks* (%)	*Ka/Ks*	π (%)	*Ka* (%)	*Ks* (%)	*Ka/Ks*	π (%)	*Ka* (%)	*Ks* (%)	*Ka/Ks*
2–10	0.234	0.194	0.391	0.497	0.613	0.489	1.091	0.448	0.846	0.684	1.503	0.455
11–20	0.418	0.358	0.668	0.536	0.683	0.58	1.095	0.53	1.164	0.995	1.899	0.524
21–30	0.618	0.496	1.07	0.464	0.802	0.626	1.44	0.435	1.446	1.18	2.583	0.457
31–40	0.542	0.456	0.899	0.507	0.672	0.556	1.129	0.492	1.26	1.074	2.091	0.513
41–50	0.618	0.543	0.945	0.574	0.661	0.533	1.152	0.463	1.384	1.17	2.319	0.505
51–60	0.653	0.533	1.087	0.49	0.703	0.539	1.098	0.491	1.209	1.035	2.013	0.514
61–70	0.547	0.479	0.849	0.564	0.444	0.384	0.685	0.561	0.925	0.775	1.556	0.498
71–79	0.696	0.566	1.232	0.46	0.404	0.295	0.815	0.363	0.869	0.751	1.39	0.54
average	0.541	0.26	0.52	0.502	0.623	0.45	1.05	0.426	1.138	0.958	1.919	0.499

In addition, the mean value of Tajima’s *D* [29] was −0.96 among the intact alleles in the *Ψ* loci, which was consistent with a previous study showing an excess of low-frequency polymorphisms in *A. thaliana* populations [30]. However, the distribution of Tajima’s *D* among their disrupted alleles had a significant deviation from negative toward zero (Figure 5), also suggesting ongoing accumulation of neutral mutations in these *Ψ* sequences.

In the *Ψ* loci, disrupted alleles were expected to evolve faster than their corresponding intact alleles. Indeed, larger *Ka* (0.0085 vs. 0.0081, *P* = 0.01) and *Ka/Ks* (0.64 vs. 0.60, *P* = 0.04) were found among disrupted alleles than that among intact alleles in centromere regions (Table 3), whereas little differentiation was observed in *Ks* (0.0133 vs. 0.0134, *P* = 0.89). On the other hand, significantly larger *Ka/Ks* also was found in centromere regions than that in telomere regions in *Ψ* loci (Table 3; *P*<0.001). However, no significant difference was detected between these two regions in non-*Ψ* loci (0.24 vs. 0.23, *P* = 0.86). A significantly negative correlation between *Ka/Ks* and the local gene densities also was detected in *Ψ* loci (*r* = 0.38 and 0.66, *P*<0.001; Figure 4G and 4H), whereas a slightly negative correlation was found in non-*Ψ* loci (*r* = 0.28, *P* = 0.002; Figure 4I).

### Functional Bias of Ψ Loci in A. thaliana Genomes

If we assume that the probability of pseudogenization is equal at every gene in each accession. The expected proportion of shared *Ψ*s between any two accessions should be 1.26% (from 10,000 times’ random repeats; Table S5). Interestingly, our observed shared *Ψ*s (17.4%) is significantly greater than the null (*P*<0.0001), suggesting that functional bias of *Ψ*s might exist.

**Figure 6:**
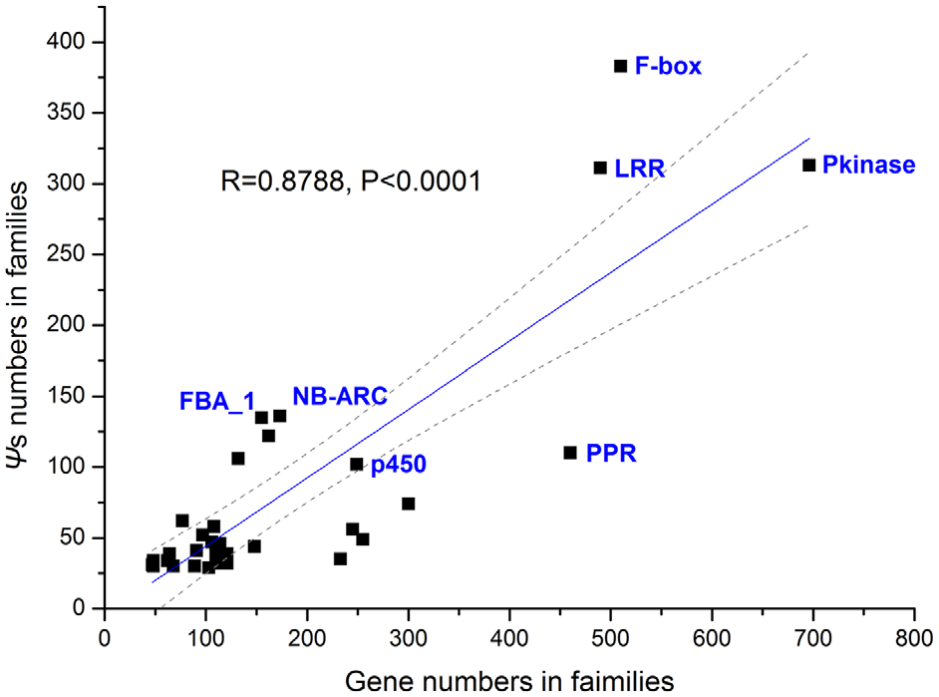
Correlation between numbers of *Ψ* loci and all ORF members in their domain families. Gray dash lines indicate the 95% confidence interval around the regression line.

**Figure 7:**
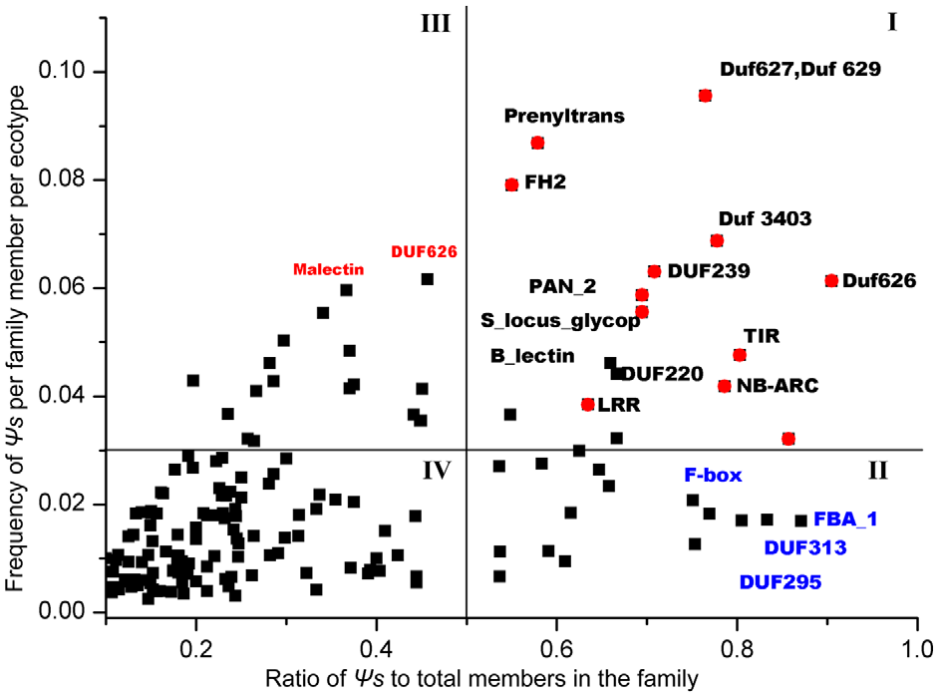
Domain families divided into four distinct categories according to the frequency of *Ψ* loci in their gene domain families (PPD, X-axis) and the average frequency of disrupted alleles (FDA, Y-axis) among the accessions in each gene domain family (also see **table 5**). Using the top 1% distribution of these two parameters as a cut-off (marked by vertical and horizontal lines), the total of 177 domain families were divided into four distinct regions (I, II, III and IV).

To further determine whether these *Ψ* loci have a functional bias, they were classified into domain families based on Pfam, domain designations using their annotated ORFs in the reference genome as queries (Table 5). Based on these domain family assignments, a significant positive correlation was detected between the number of *Ψ* loci and that of all ORF members in their domain families (Figure 6; r = 0.88, *P*<0.0001), consistent with previous reports [10]. This result indicates that larger domain families likely have proportionally higher numbers of *Ψ* loci. However, many domain families seemed to show large deviations from the trend line in Figure 6. These domain families also exhibited different frequencies of the disrupted alleles among the accessions (Table 5). Thus, the proportion of *Ψ* loci in the domain family (PPD) and their average frequency of the disrupted alleles (FDA) among the 80 accessions may be good parameters for addressing which domain families have an overrepresented number or frequency of *Ψ*s (Table 5). Using the top 1% distribution of these two parameters as a cut-off, 177 domain families in total were divided into four distinct regions (I, II, III and IV; Figure 7). Region I contained 17 gene domain families and had both the highest PPD and FDA (Figure 7 and Table 5), including many common domain families in plants, e.g. *NB-ARC*, *TIR*, *LRR*, *S_locus_glycop*, *B_lectin*, *PAN_2*, and *MATH*. In regions II and III, other 17 gene domain families were detected in each region with the top 1% of either PPD or FDA, including *Pkinase*, *F-box*, *P450*, *self-incomp_S1*, *FBA_1*, *terpene_synth* and *DEAD* domain families.

**Table 5:** Frequency of *Ψ*s in domain families.

Domain	*Ψ*s	Gene No.in Col.	Average frequency of disrupted allelesin the 80 ecotypes	Frequency of *Ψ* loci	Frequency of *Ψ*s per ecotype
F-box	383	510	2.21	0.751	0.021
Pkinase	313	696	6.31	**0.450**	**0.035**
LRR	311	490	4.85	**0.635**	**0.038**
NB-ARC	136	173	4.26	**0.786**	**0.042**
FBA_1	135	155	1.56	0.871	0.017
C1_3	122	162	1.34	0.753	0.013
PPR	110	460	2.21	0.239	0.007
TIR	106	132	4.74	**0.803**	**0.048**
p450	102	249	2.95	0.410	0.015
zf-C3HC4	74	300	3.35	0.247	0.010
DUF295	62	77	1.69	0.805	0.017
Kelch_1	58	108	1.68	0.537	0.011
RRM_1	56	245	7.58	0.229	0.022
DUF26	52	97	4.04	0.536	0.027
Myb_DNA-binding	49	255	3.77	0.192	0.009
SRF-TF	47	106	3.21	0.443	0.018
UDPGT	46	114	1.53	0.404	0.008
Helicase_C	44	148	13.54	0.297	0.050
B3	41	91	7.35	0.451	0.041
Self-incomp_S1	39	64	1.25	0.609	0.010
Lipase_GDSL	39	110	4.72	0.355	0.021
PMEI	39	121	1.82	0.322	0.007
2OG-FeII_Oxy	38	121	4.6	0.314	0.018
WD40	35	233	10	0.150	0.019
DUF239	34	48	7.12	**0.708**	**0.063**
MATH	34	62	5.34	**0.548**	**0.037**
NAM	32	110	3.03	0.291	0.011
AAA	32	121	9.61	0.264	0.032
B_lectin	31	47	5.59	**0.660**	**0.046**
Jacalin	30	48	3.83	0.625	0.030
Terpene_synth_C	30	68	6.64	0.441	0.037
Abhydrolase_1	30	89	5.18	0.337	0.022
DEAD	29	103	13.1	0.282	0.046

Notably, most of the domain families were related to adaptive responses to environmental stimuli and biotic stress, e.g. defense proteins: *NBS-LRR* proteins (including *NB-ARC*, *TIR* and *LRR* domains), *SRK* proteins (including *S_locus_glycop*, *B_lectin* and *PAN_2* domains), RLK proteins (including *LRR* and *Pkinase* domains); biotic or abiotic stress response proteins: *F-box* proteins, cytochrome *P450*, F-box associated proteins (*FBA_1*) and terpene synthase (*terpene_synth*). As expected, the observed shared *Ψ*s in these domain families are significantly greater than the null (from randomization: *P*<0.0001; Table S5), also suggesting a clear functional bias in these *Ψ* loci. On the other hand, the shared *Ψ*s are not more than 30% between any two accessions either in the whole genome or in these domain families (Table S5), suggesting that such a high number of *Ψ*s may play a crucial role in phenotypic diversities between accessions and these shared *Ψ*s frequently involved in stress responses have an elevated mutation rate, likely providing a pool of highly dynamic targets for selection to mediate interactions with the ever-changing environments.

## Discussion

### Adaptive Evolution of Arabidopsis Contribute to the Bias of Ψs

Previous studies focusing on the duplicated or retrotransposed *Ψ*s have shown that they are ubiquitous and abundant in eukaryotic genomes [31]. Using a homology-based approach, ∼8000 retrotransposed and ∼3000 duplicated *Ψ*s were detected in the human genome draft [8]. Similarly, ∼2700 and 5600 *Ψ*s were found in *A. thaliana* and rice genomes [10], [18]. However, few studies have investigated the dynamics of pseudogenization in alleles within species. Most recently, high-density array resequencing in 20 diverse *A. thaliana* accessions showed that 1614 genes harbor at least one large-effect SNP, including a premature stop codon, a frameshift mutation or a shift in intron-exon structure in at least one accession [13]. Similarly, large-effect SNPs were detected in 4648 soybean genes in 31 resequenced wild and cultivated soybean genomes [32].

In this study, a systematic comparative analysis was conducted of *Ψ*s within the 80 fully re-sequenced *A. thaliana* accessions using 27,133 annotated protein-coding genes in Col-0 as references. Disruptive mutations were found in ∼28% of the annotated CDSs among the 80 re-sequenced accessions (Table 1), which is consistent with the recent report on *Ψ*s among these 80 accessions [17] and 18 other accessions [14]. An average of ∼930 *Ψ*s was detected in each accession, and each *Ψ* was present in an average of ∼6 accessions, suggesting that a remarkable proportion of *Ψ*s is maintained among *A. thaliana* populations. On the other hand, based on the analysis of functional categories, there is a clear functional bias in these *Ψ* genes, which are mainly involved in responses to environmental stimuli and biotic stress, suggesting that they are likely important for adaptive evolution to rapidly changing environments. For example, 136 out of 173 NBS genes, 311 out of 490 LRR genes, 102 out of 249 P450 genes, and 383 out of 510 F-box genes had allelic *Ψs* in the 80 accessions.

Since different accessions have very different life histories, the nature of selective pressure imposed by their environmental conditions is expected to be diverse [17], [33]. Therefore, it is reasonable that *Ψ* loci tend to be involved in responses to biotic stress and environment stimuli. For example, it is clear that *A. thaliana* plants can defend against a wide array of pathogens, yet there is great variability in those resistance genes for such defenses, indicating extensive environment-dependent variation. On the one hand, the fitness costs associated with individual *R*-genes have been observed frequently in field trials, e.g. *RPM1* (a NBS-LRR gene) [34] and *ACD6* (At4g14400, enhances resistance to a broad range of pathogens [35] and was detected as a *Ψ* in 7 accessions), which may be a possible explanation for the frequent pseudogenization in these genes.

On the other hand, *Ψ*s are expected to be evolving neutrally and have higher levels of nucleotide diversity than other loci. Therefore, new alleles can be continuously generated in their population. Normally, some *Ψ*s can disappear with time by the accumulation of successive mutations, while some *Ψ*s with alterations may be repaired by reverse mutations, gene conversion or reactivation by translational recording events [2], [36], [37]. Therefore, the high mutations in these *Ψ*s likely provide a pool of highly dynamic targets for selection in ever-changing environments. Obviously, the *Ψ* variations may be a possible mechanism for phenotypic differentiation reflecting evolutionary adaptation of the species to the different habitats and environmental pressures.

### Natural Selection Contributes to the Regional Distribution of Ψs

*Ψ*s have long been assumed to be evolving neutrally. Indeed, Torrents and colleagues [38] have demonstrated that approximately 95% of the *Ψ*s in the human genome are evolving neutrally. Under the neutral evolutionary scenario, the gene length may play an important role in the duplicative pseudogenization: longer genes should be more susceptible to producing duplicative mutations as they can accomodate more deleterious mutations [39]. However, across the 80 accessions, the intact CDSs in Col-0 of these *Ψ*s showed shorter gene length, less exon number and lower GC content compared with non-*Ψ*s, especially in the centromere regions (Table 2), suggesting a deviation from the neutral evolutionary hypothesis for these *Ψ*s.

In addition, among these re-sequenced accessions, higher frequencies of *Ψs* and higher levels of nucleotide diversity were detected in the gene desert regions (Figure 3A and Figure 4). Higher levels of nucleotide diversity, especially of nonsynonmous substitutions, also were found in the *Ψ* loci in these regions (Figure 4) by pairwise comparison of *Ψ* sequences in some accessions and the intact alleles in the other accessions (Table 3). However, as mentioned above, these *Ψ*s have a clear functional bias mainly involving responses to environmental stimuli and biotic stress (Figure 7), suggesting that these genes are likely important for adaptive evolution to rapidly changing environments. These results indicate: (i) a markedly nonrandom distribution of *Ψ*s across the genome, with a regional preference for the gene desert regions; (ii) *Ψ*s have a clear functional bias mainly involving responses to environmental stimuli and biotic stress; (iii) and higher levels of nucleotide diversity in the gene desert regions, including *Ψ* loci and other functional genes.

Generally, genes mediating stress responses need to accumulate more nucleotide substitutions to respond to the rapidly changing environments [36], [40], [41]. On the other hand, in the gene desert regions, the high nucleotide diversity of genes is not due to lack of selective constraint, but it is possible that there are few targets for purifying or positive selection to appreciably reduce diversity below that in the gene-rich regions [27]. Therefore, a possible explanation for the regional bias of these *Ψ*s may be natural selection rather than random distribution.

### Natural Selection is Supported by the Clear Population Structure of Ψs

**Figure 8:**
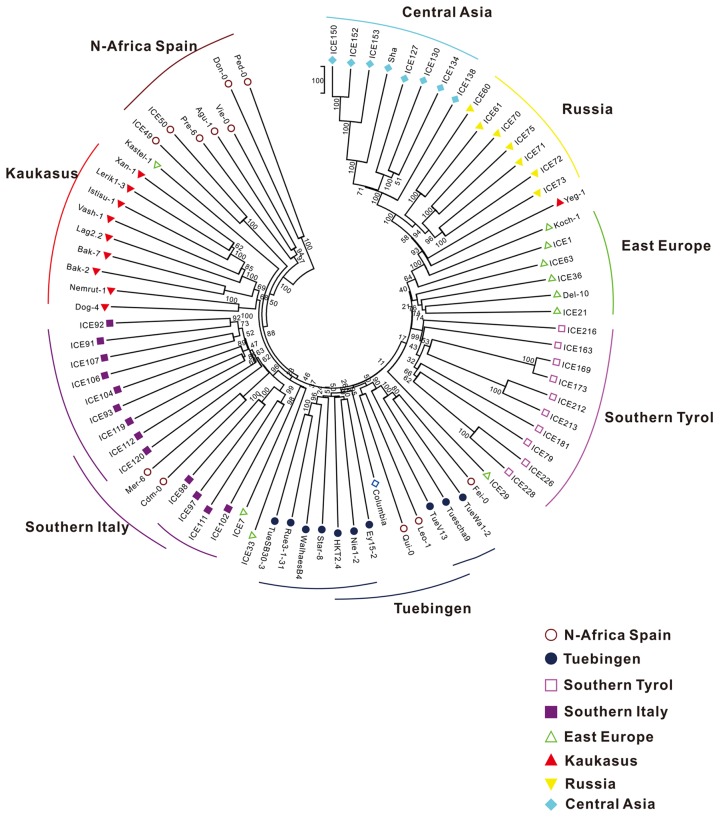
Phylogenetic tree based on geographic structure of *A. thaliana* accessions.

The 80 re-sequenced accessions were collected from eight geographic regions of Eurasia: the Iberian Peninsula with North Africa, Southern Italy, Eastern Europe, the Caucasus, Southern Russia, Central Asia, Swabia (in the southwest of Germany) and South Tyrol (in the north of Italy) [17]. Theoretically, the distribution of *Ψ*s should have a significant positive correlation with the geographic structure of these accessions. Therefore, a phylogenetic tree (Figure 8) was constructed based on genome-wide *Ψ* polymorphisms in the 80 re-sequenced accessions by the discrete morphology (parsimony) method using the PARS programs of the PHYLIP package v3.6. As expected, most of the accessions from the same region clustered in the same clade (Figure 8), indicating that they shared more common *Ψ*s. For example, the average shared *Ψs* between any two accessions are 17.5% and 29.6% in North Africa and Central Asia, respectively, which are significant larger than that (14.8%) between these two regions (*P*<0.001). This finding also suggests that a clear population structure influenced the distribution of *Ψ*s. Since *Ψ*s have a clear functional bias for genes involved in responses to environmental stimuli and biotic stress, and higher diversities were detected in these *Ψ* loci, it is clear that the local adaptation to divergent environments may lead to dramatic differences in the distribution of *Ψ*s between populations. Such variation also offers particularly compelling evidence of the natural selection on *Ψ*s when correlated with variations in environmental factors over multiple independent geographic regions [42], [43].

## Supporting Information

Figure S1 **Frequency distribution of the frameshift or premature alleles in the 80 re-sequenced accessions.**
(PDF)

Figure S2 **Distribution of divergences (** ***Ka*** **and** ***Ks*** **) between** ***Ψ*** **s with increasing frequency (2 to 10 ecotypes) of disrupted alleles.** X-axis: frequency (2–10) of disrupted alleles; Y-axis: average Ka and Ks among a group of alleles. Black dots and line for Ka, and red dots and line for Ks. A) Ka and Ks for disrupted alleles, B) Ka and Ks for intact alleles.
(PDF)

Table S1 Number of identified *Ψ* loci in each accession of *A. thaliana*.
(PDF)

Table S2 Frequency of disrupted alleles in 80 re-sequenced *A. thaliana* accessions.
(PDF)

Table S3 Distribution of *Ψ* loci on the five chromosomes of *A. thaliana*.
(PDF)

Table S4 Distribution of *Ψ* loci in telomere and centromere regions.
(PDF)

Table S5 Observed and expected proportion of shared *Ψ*s in genomes or gene families between any two accessions.
(PDF)
