# Supplementary material for: Genome-Wide Survey of Pseudogenes in 80 Fully Re-sequenced Arabidopsis thaliana Accessions
Source: PLoS One. 2012 Dec 13;7(12):e51769. doi: 10.1371/journal.pone.0051769 (PMC3521719; doi:10.1371/journal.pone.0051769)

Figure S1. Frequency distribution of the frameshift or premature alleles in the 80 re-sequenced accessions

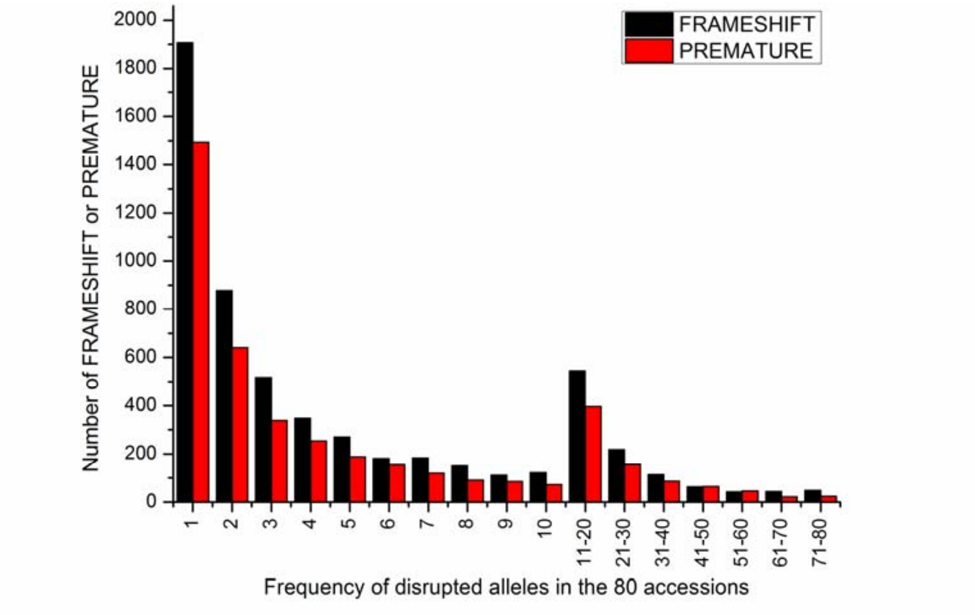

Supplement: Figure S1 — Frequency distribution of the frameshift or premature alleles in the 80 re-sequenced accessions. (PDF) [file pone.0051769.s001.pdf]
