# Supplementary material for: Genome-Wide Survey of Pseudogenes in 80 Fully Re-sequenced Arabidopsis thaliana Accessions
Source: PLoS One. 2012 Dec 13;7(12):e51769. doi: 10.1371/journal.pone.0051769 (PMC3521719; doi:10.1371/journal.pone.0051769)

Figure S2. Distribution of divergences (Ka and Ks) between *Ψ*s with increasing frequency (2 to 10 ecotypes) of disrupted alleles. X-axis: frequency (2-10) of disrupted alleles; Y-axis: average Ka and Ks among a group of alleles. Black dots and line for Ka, and red dots and line for Ks. A) Ka and Ks for disrupted alleles, B) Ka and Ks for intact alleles.

A

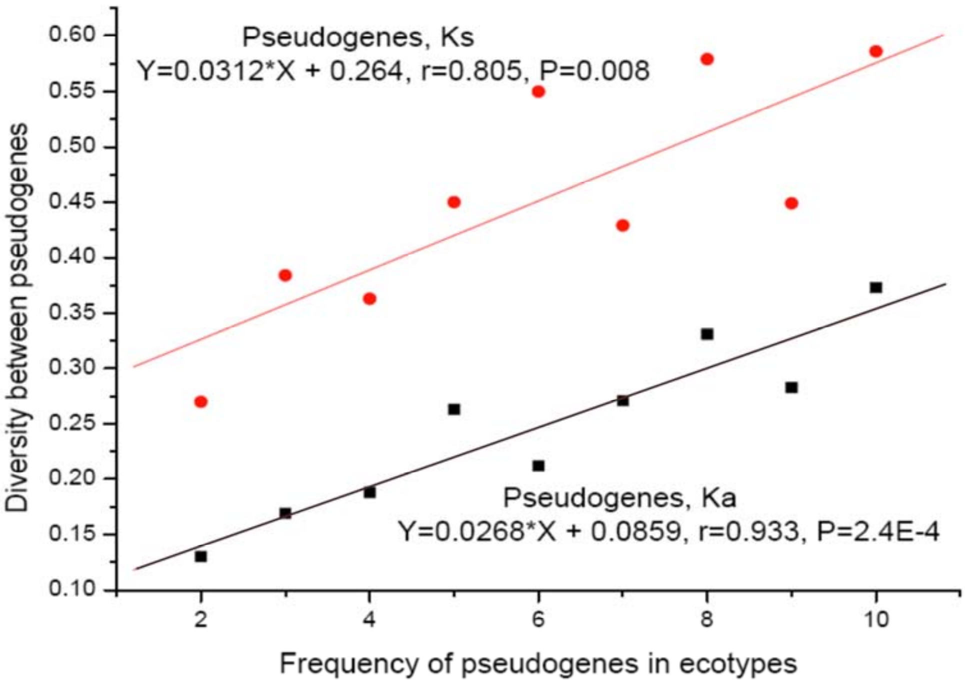

B

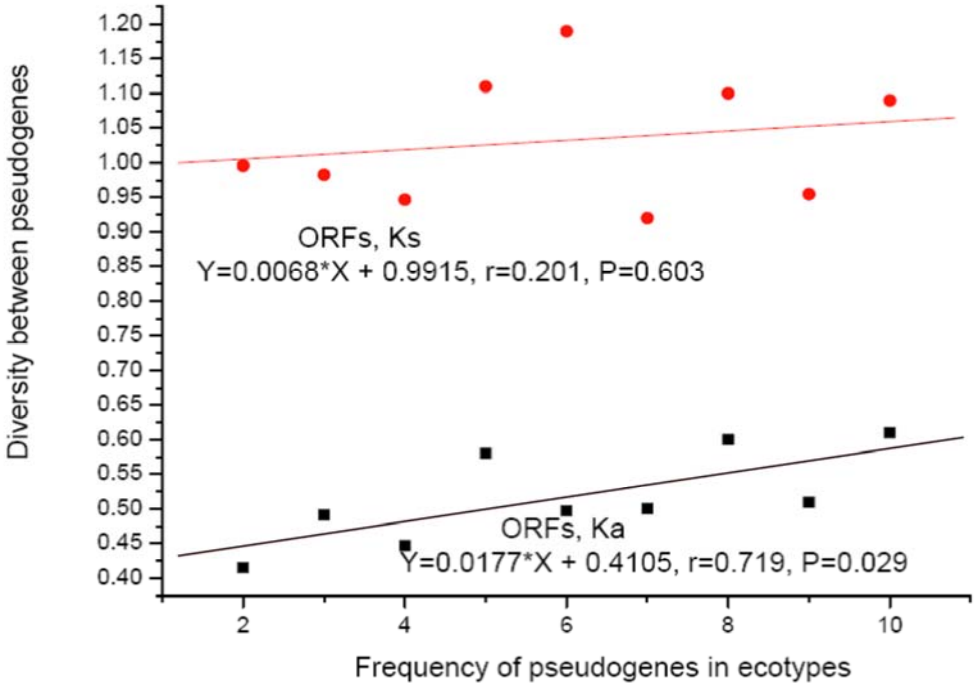

Supplement: Figure S2 — Distribution of divergences ( Ka and Ks ) between Ψ s with increasing frequency (2 to 10 ecotypes) of disrupted alleles. X-axis: frequency (2–10) of disrupted alleles; Y-axis: average Ka and Ks among a group of alleles. Black dots and line for Ka, and red dots and line for Ks. A) Ka and Ks for disrupted alleles, B) Ka and Ks for intact alleles. (PDF) [file pone.0051769.s002.pdf]
