# Supplementary material for: Genome-Wide Survey of Pseudogenes in 80 Fully Re-sequenced Arabidopsis thaliana Accessions
Source: PLoS One. 2012 Dec 13;7(12):e51769. doi: 10.1371/journal.pone.0051769 (PMC3521719; doi:10.1371/journal.pone.0051769)
Supplement: Table S1 — Number of identified Ψ loci in each accession of A. thaliana. (PDF) [file pone.0051769.s003.pdf]

Table S1. Number of identified *Ψ* loci in each accession of *A. thaliana*

Accession name	Region	Pseudogene number (3/3)
Col-0	Columbia	(reference)
Rue3-1-31	Tuebingen	794
WalhaesB4	Tuebingen	795
TueV13	Tuebingen	801
TueSB30-3	Tuebingen	811
Nie1-2	Tuebingen	830
Star-8	Tuebingen	830
ICE163	Southern Tyrol	833
ICE92	Southern Italy	836
Tuescha9	Tuebingen	849
Ey15-2	Tuebingen	850
ICE36	East Europe	855
Fei-0	N-Africa Spain	855
ICE21	East Europe	866
ICE63	East Europe	871
HKT2.4	Tuebingen	881
ICE102	Southern Italy	881
Dog-4	Kaukasus	884
ICE1	East Europe	886
Del-10	East Europe	888
ICE98	Southern Italy	889
Nemrut-1	Kaukasus	897
Leo-1	N-Africa Spain	898
Qui-0	N-Africa Spain	899
ICE73	Russia	901
Bak-7	Kaukasus	902
TueWa1-2	Tuebingen	903
Koch-1	East Europe	903
ICE7	East Europe	906
Vie-0	N-Africa Spain	914
ICE91	Southern Italy	916
ICE228	Southern Tyrol	919
ICE112	Southern Italy	920
ICE104	Southern Italy	921
ICE29	East Europe	921
ICE216	Southern Tyrol	922
ICE61	Russia	924
ICE120	Southern Italy	926
ICE119	Southern Italy	928

ICE107	Southern Italy	931
ICE127	Central Asia	933
ICE181	Southern Tyrol	934
Cdm-0	N-Africa Spain	935
ICE226	Southern Tyrol	936
ICE97	Southern Italy	938
ICE138	Central Asia	940
ICE106	Southern Italy	942
ICE79	Southern Tyrol	942
Kastel-1	East Europe	943
ICE152	Central Asia	948
ICE93	Southern Italy	949
ICE75	Russia	949
ICE169	Southern Tyrol	950
ICE111	Southern Italy	951
ICE212	Southern Tyrol	951
Xan-1	Kaukasus	952
ICE173	Southern Tyrol	954
ICE213	Southern Tyrol	957
Pre-6	N-Africa Spain	960
Agu-1	N-Africa Spain	962
Vash-1	Kaukasus	963
ICE33	East Europe	964
Istisu-1	Kaukasus	964
ICE71	Russia	971
Sha	Central Asia	971
Lerik1-3	Kaukasus	975
ICE60	Russia	978
Bak-2	Kaukasus	978
Lag2.2	Kaukasus	978
Mer-6	N-Africa Spain	989
ICE70	Russia	990
ICE134	Central Asia	993
ICE153	Central Asia	1000
ICE72	Russia	1005
Yeg-1	Kaukasus	1006
ICE150	Central Asia	1013
ICE50	N-Africa Spain	1018
ICE130	Central Asia	1018
ICE49	N-Africa Spain	1030
Ped-0	N-Africa Spain	1158
Don-0	N-Africa Spain	1209
