# Supplementary material for: Genome-Wide Survey of Pseudogenes in 80 Fully Re-sequenced Arabidopsis thaliana Accessions
Source: PLoS One. 2012 Dec 13;7(12):e51769. doi: 10.1371/journal.pone.0051769 (PMC3521719; doi:10.1371/journal.pone.0051769)
Supplement: Table S2 — Frequency of disrupted alleles in 80 re-sequenced A. thaliana accessions. (PDF) [file pone.0051769.s004.pdf]

Table S2. Frequency of disrupted alleles in 80 re-sequenced *A. thaliana* accessions

Frequency of disrupted alleles in the 80 accessions	Number of Ψ loci
1	3342
2	1167
3	636
4	386
5	266
6	216
7	190
8	154
9	117
10	88
11-20	512
21-30	197
31-40	113
41-50	48
51-60	54
61-70	40
71-80	56
