# Supplementary material for: Genome-Wide Survey of Pseudogenes in 80 Fully Re-sequenced Arabidopsis thaliana Accessions
Source: PLoS One. 2012 Dec 13;7(12):e51769. doi: 10.1371/journal.pone.0051769 (PMC3521719; doi:10.1371/journal.pone.0051769)
Supplement: Table S3 — Distribution of Ψ loci on the five chromosomes of A. thaliana. (PDF) [file pone.0051769.s005.pdf]

Table S3. Distribution of *Ψ* loci on the five chromosomes of *A. thaliana*

Chromosomes	no. of ORFs	Disabling mutations in the first 1/3 region		Disabling mutations in 0-2/3 region	
		no. of *Ψ*s	*Ψ*/ORF	no. of *Ψ*s	*Ψ*/ORF
1	7040	982	0.139	1446	0.205
2	4233	608	0.144	887	0.210
3	5429	797	0.147	1182	0.218
4	4122	607	0.147	913	0.221
5	6309	842	0.133	1271	0.201
Total (or average)	27133	3836	0.142 ±0.006	5699	0.211 ±0.008
