# Supplementary material for: Genome-Wide Survey of Pseudogenes in 80 Fully Re-sequenced Arabidopsis thaliana Accessions
Source: PLoS One. 2012 Dec 13;7(12):e51769. doi: 10.1371/journal.pone.0051769 (PMC3521719; doi:10.1371/journal.pone.0051769)
Supplement: Table S4 — Distribution of Ψ loci in telomere and centromere regions. (PDF) [file pone.0051769.s006.pdf]

Table S4. Distribution of *Ψ* loci in telomere and centromere regions

Chromosomes	Disabling mutations in the first 1/3 region			Disabling mutations in 0- 2/3 region		
	Ψ/ORF		Telomere vs. Centromere	*Ψ*/ORF		Telomere vs. Centromere
	Telomere	Centromere	*t*-test	Telomere	Centromere	*t*-test
1	0.086	0.321	0.0004***	0.127	0.432	1.45E-5***
2	0.087	0.339	0.042*	0.136	0.426	0.025*
3	0.100	0.407	4.87E-6***	0.158	0.561	3.81E-4***
4	0.079	0.308	0.0042**	0.132	0.436	0.002**
5	0.094	0.317	8.75E-8***	0.148	0.455	0.0010***
Average	0.0892 ±0.008	0.338 ±0.040	8.34E-11***	0.140±0.013	0.462 ±0.056	1.2E-11***

Numbers of *Ψ* loci and ORFs were calculated in 4Mb from the tips of the chromosomes (Telomere) or around the centromere (Centromere) in each chromosome.
