# Supplementary material for: Genome-Wide Survey of Pseudogenes in 80 Fully Re-sequenced Arabidopsis thaliana Accessions
Source: PLoS One. 2012 Dec 13;7(12):e51769. doi: 10.1371/journal.pone.0051769 (PMC3521719; doi:10.1371/journal.pone.0051769)
Supplement: Table S5 — Observed and expected proportion of shared Ψs in genomes or gene families between any two accessions. (PDF) [file pone.0051769.s007.pdf]

Table S5. Observed and expected proportion of shared *Ψ*s in genomes or gene families between any two accessions.

	Observations (%)	Simulations (from10,000 times' random repeats) (%)	*P* values between Observations and Simulations
Proportion of shared *Ψ*s between any two accessions	17.4 ± 2.8	1.26	<0.0001
Proportion of shared *NB-ARC Ψ*s between any two accessions	16.1 ± 5.2	5.15	<0.0001
Proportion of shared *LRR Ψ*s between any two accessions	15.3 ± 4.4	4.31	<0.0001
Proportion of shared *TIR Ψ*s between any two accessions	19.3 ± 5.6	5.66	<0.0001
Proportion of shared *Pkinase Ψ*s between any two accessions	16.1 ± 4.7	3.36	<0.0001
Proportion of shared *F-box Ψ*s between any two accessions	18.2 ± 4.1	5.61	<0.0001
Proportion of shared *P450 Ψ*s between any two accessions	14.9 ± 7.1	4.41	<0.0001
Proportion of shared *PPR Ψ*s between any two accessions	27.1 ± 4.9	5.96	<0.0001
Proportion of shared *C1_3 Ψ*s between any two accessions	18.5 ± 5.7	5.93	<0.0001
Proportion of shared *FBA_1 Ψ*s between any two accessions	16.8 ± 5.7	6.29	<0.0001

Simulations: we randomly sample *x* genes from the genome-wide or the gene families in each *A.thaliana* accession (*x* is equal to the number of observed *Ψ*s in genome or gene families in the accession). Then, we can calculate the expected proportion of shared genes between any two accessions. This process was repeated 10,000 times.
